# Lateness of Acetaminophen and Non-steroidal Anti-inflammatory Drug Administrations in a Retrospective Cohort of Medication Administration Records Among Patients After Cesarean Delivery

**DOI:** 10.7759/cureus.61433

**Published:** 2024-05-31

**Authors:** Unyime S Ituk, Franklin Dexter, Katherine E Hadlandsmyth

**Affiliations:** 1 Department of Anesthesia, University of Iowa, Iowa CIty, USA; 2 Department of Anesthesia, University of Iowa, Iowa City, USA

**Keywords:** hospital engineering, industrial engineering, operations research, non-opioid analgesia, medication administration record, anesthesia for cesarean delivery

## Abstract

Introduction: In an earlier study of patients after cesarean delivery, the concurrent versus alternating administration of acetaminophen and non-steroidal anti-inflammatory drugs was associated with a substantial reduction in total postoperative opioid use. This likely pharmacodynamic effect may differ if the times when nurses administer acetaminophen and non-steroidal anti-inflammatory drugs often differ substantively from when they are due. We examined the "lateness" of analgesic dose administration times, the positive difference if administered late, and the negative value if early.

Methods: The retrospective cohort study used all 67,900 medication administration records for scheduled (i.e., not "as needed") acetaminophen, ibuprofen, and ketorolac among all 3,163 cesarean delivery cases at the University of Iowa between January 2021 and December 2023. Barcode scanning at the patient's bedside was used right before each medication administration.

Results: There were 95% of doses administered over a 4.8-hour window, from 108 minutes early (97.5% one-sided upper confidence limit 105 minutes early) to 181 minutes late (97.5% one-sided lower limit 179 minutes late). Fewer than half of doses (46%, P <0.0001) were administered ±30 minutes of the due time. The intraclass correlation coefficient was approximately 0.11, showing that there were small systematic differences among patients. There likewise were small to no systematic differences in lateness based on concurrent administrations of acetaminophen and ibuprofen or ketorolac, time of the day that medications were due, weekday, year, or number of medications to be administered among all such patients within 15 minutes.

Discussion: Other hospitals should check the lateness of medication administration when that would change their ability to perform or apply the results of analgesic clinical trials (e.g., simultaneous versus alternating administration).

## Introduction

Acetaminophen and non-steroidal anti-inflammatory drugs (NSAID) are integral components of multimodal postoperative analgesia. Forkin et al. performed a prospective cohort study of patients recovering from cesarean delivery [1]. The concurrent administration of acetaminophen and NSAID was associated with a substantial reduction in total postoperative opioid use versus alternating administration [1]. Their study was novel because earlier studies made comparisons of single drugs versus both or versus placebo, not administration timing [2,3].

For the evaluation of concurrent versus alternating administration in a randomized controlled trial, the timing of medication administration would affect the likely pharmacodynamic effect [1] of interest. Designing such a trial would be challenging if the times when medications were administered often differed substantively from when they were due based on physicians' orders. "Lateness" is a difference, positively valued if a drug is administered late and negatively valued if administered early [4]. There is a dearth of studies analyzing medication administration records to estimate lateness for any category of medications. PubMed, Scopus, and Google Scholar searches were performed on 3 May 2024. The searches found no related articles (https://FDshort.com/ItukDexterHadlandsmyth). The United States Centers for Medicare & Medicaid Services requires its hospitals to have a policy for the "timeliness of medication administration" [5]. University of Iowa Health Care's policy is that "time-critical scheduled medications must be administered within 30 minutes before or after the scheduling (administration) time" [6]. Analgesics are not included in the categories of time-critical medications (i.e., the policy on the timeliness of nursing administration of analgesics is that there are no requirements) [6].

For the current study, our goal was to quantify the lateness [4] of administrations covering 95% of the population of acetaminophen [1] and NSAID [1] doses among our patients who had cesarean delivery [1] from 2021-2023. In other words, we estimated the interval of medication times excluding the 2.5% that had earlier administration time and the 2.5% that had later administration time. We tested whether the lateness would exceed ±30 minutes [5,6] (i.e., one-hour interval) for most (>50%) doses administered.

## Materials and methods

The medication administration records for all 3,163 cesarean delivery cases (among 3,047 patients) done at the University of Iowa between 1 January 2021 and 31 December 2023 were included (Table 1). Barcode scanning at the patient's bedside was used before all acetaminophen, ibuprofen, and ketorolac administrations. (These are the three analgesics used routinely for these patients.) Variables from the medication administration records used in analyses were medication, due date/time, administered date/time, and blinded patient identifier. The 5858 "as needed" doses of the three medications were not included except in the last seven rows of (Table 1), for the counts of other analgesics due within ± 15 min.

**Table 1:** Difference in minutes between administered times and due times of acetaminophen, ibuprofen, or ketorolac that were scheduled doses (i.e., not “as needed”) SD represents the standard deviation in minutes. The “count other analgesics due within ±15 minutes” means among all the obstetrical patients with cesarean delivery, including the analgesics administered as needed.

Category	Mean	SD	Count	5%	10%	25%	50%	75%	90%	95%
All administrations	25	79	67,900	-76	-48	-11	15	59	112	149
Acetaminophen; when NSAID due ±15 min	23	81	5,813	-76	-54	-13	14	58	110	146
Acetaminophen; when NSAID not due ±15 min	22	80	35,407	-80	-54	-13	14	58	110	146
Acetaminophen; when NSAID due ±30 min	23	85	9,278	-78	-54	-13	14	58	110	146
Acetaminophen; when NSAID not due ±30 min	23	78	31,942	-80	-54	-13	14	58	110	146
Medication due 00:00-03:59	27	73	11,383	-75	-47	-11	15	62	121	156
Medication due 04:00-07:59	22	72	11,649	-78	-50	-11	13	54	105	142
Medication due 08:00-11:59	26	82	12,195	-76	-47	-9	17	60	113	149
Medication due 12:00-15:59	27	78	10,928	-79	-49	-12	17	62	118	157
Medication due 16:00-19:59	24	81	10,468	-73	-47	-11	15	56	106	141
Medication due 20:00-23:59	24	90	11,277	-74	-46	-12	13	57	110	145
Sunday	27	88	8,453	-83	-54	-12	17	65	122	160
Monday	23	76	7,598	-76	-50	-13	13	57	110	147
Tuesday	25	80	8,962	-67	-43	-11	14	55	107	145
Wednesday	23	76	10,235	-75	-48	-12	13	58	113	146
Thursday	23	75	10,896	-74	-48	-11	14	56	106	144
Friday	24	75	11,275	-78	-47	-10	15	58	110	148
Saturday	29	87	10,481	-78	-46	-8	19	62	117	154
Year 2021	25	83	21,055	-78	-49	-11	14	58	112	148
Year 2022	25	77	22,432	-75	-46	-10	15	58	110	147
Year 2023	25	78	24,413	-75	-49	-12	16	60	115	151
Count other analgesics due within ±15 min: 01	24	76	14,775	-79	-51	-13	16	60	112	146
Count other analgesics due within ± 15 min: 02	24	78	19,967	-76	-49	-12	15	58	111	148
Count other analgesics due within ± 15 min: 03	26	84	15,753	-76	-47	-11	15	60	115	151
Count other analgesics due within ± 15 min: 04	25	84	9,252	-74	-45	-9	15	57	111	149
Count other analgesics due within ± 15 min: 05	26	76	4,789	-73	-43	-9	15	58	116	155
Count other analgesics due within ± 15 min: 06	24	68	2,051	-71	-47	-9	13	51	113	150
Count other analgesics due within ± 15 min: 07	26	80	914	-64	-43	-9	13	56	110	147

Percentiles were estimated using all N =67,900 medication administration records. The two-sided 95% prediction interval for lateness was calculated using the exact binomial method (Stata v18.0 centile cii command; StataCorp, College Station, Texas) [7-9]. The prediction interval is centered around zero because, from above, lateness is positively valued when administered late and negatively valued when administered early [4]. The 97.5% upper confidence limit for the 2.5th percentile was used to define the prediction interval's lower bound, and the 97.5% lower confidence limit for the 97.5% percentile was used to define the prediction interval's upper bound. This ensured a greater than 5% probability that the true lateness limits fell outside the reported range. Percentiles of minutes late were estimated among the potential covariates in Table 1 using the R-6 interpolation method (Microsoft Excel PERCENTILE.EXC function) [10]. The lag-1 Kendall tau-b correlation of lateness was calculated by sorting records by the due date/times while treating multiple medications of the same patient with the same due date/time and administration date/time as one lateness value.

The exact binomial probability test was used to compare the observed percentage lateness ±30 minutes to 50%. There was >99% statistical power to detect a 1% difference, treating p<0.05 as statistically significant.

**Figure 1:**
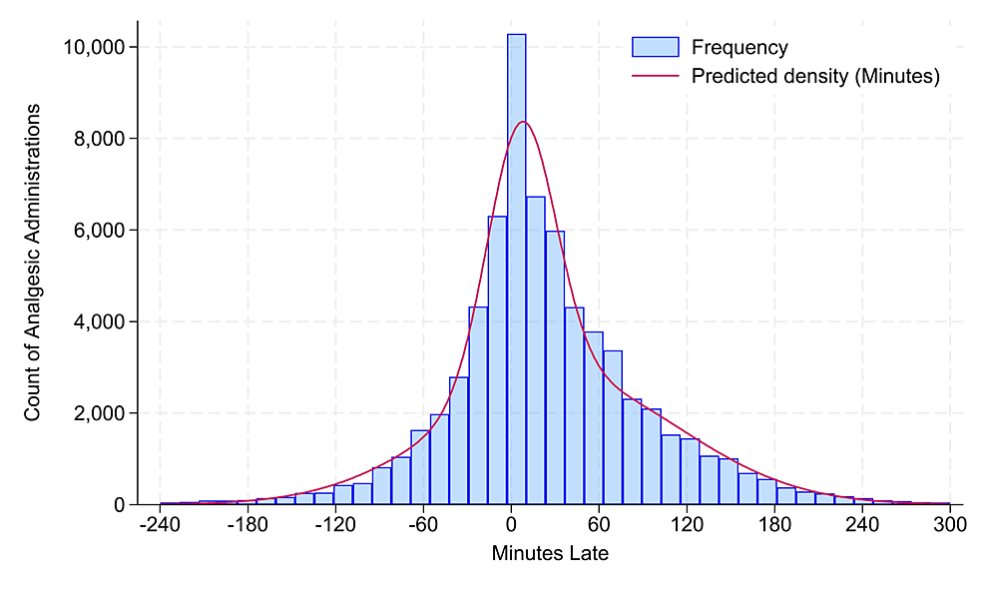
Histogram of the lateness of analgesic administration relative to due times The sample size N = 67,731. From the original 67,900, there were 22 dropped with lateness less than 240 minutes (4.0 hours), and 147 dropped with lateness longer than 300 minutes (5.0 hours). There was approximately 38% of the population drawn from a normal distribution with a mean lateness of 7 minutes and a standard deviation of 23 minutes. The remaining approximately 62% was drawn from a second normal distribution with a mean of 34 minutes and a standard deviation of 81 minutes. There was an overall approximately 46% of administrations ±30 minutes of the due time because of the 38% centered around that time plus an extra component of times from the second curve that happened to be administered earlier than usual.

Parametric analyses were performed after dropping the 0.2% outliers exceeding 4.0 hours early and 5.0 hours late (Figure 1). The frequency distribution curve in (Figure 1) was created among doses using the Stata fmm and estat lcprob commands, fitting a mixture of two normal distributions. The variability among patients was examined using the intraclass correlation coefficient, calculated using the Stata loneway command for one-way random effects analysis-of-variance. Three years of data were chosen to have a sufficient sample size to study each weekend day (e.g., 156 Sundays), even if there was a moderately large (e.g., >0.35) intraclass correlation coefficient (Table 1) [4].

## Results

Among the 67,900 analgesics doses, there were 61% acetaminophen (41,220), 24% ibuprofen (16,148), and 16% ketorolac (10,532). The probability distribution of lateness is a mixture of two distributions (Figure 1). There were 95% of doses administered between 108 minutes early (1.8 hours, 97.5% one-sided upper confidence limit 105 minutes early) and 181 minutes late (3.0 hours, 97.5% one-sided lower confidence limit 179 minutes late). Fewer than half of the doses (P <0.0001) were administered ±30 minutes of the due time (46%, 31,435). The lag-1 Kendall tau-b correlation based on due date/times was 0.014 (N =65,146, P <0.0001). The estimated intraclass correlation coefficient was 0.11 (95% confidence interval 0.10 to 0.11), showing that there were small systematic differences among patients. There likewise were small to no systematic differences based on concurrent administrations of acetaminophen and ibuprofen or ketorolac, hour of the day that medications were due, weekday, year, or number of medications to be administered among all such patients within 15 minutes (Table 1). For example, when scrolling among rows of the median (50th percentile) column, the maximum observed pairwise differences among categories are six minutes for weekdays.

As a control, the analgesic doses had a mean (standard deviation) lateness of administration of 25 (79) minutes (N = 67,900, Table 1). The subset of 475/3163 cases and 471/3047 patients receiving postoperative antibiotics had a similar mean lateness of those administrations, 26 (84) minutes (N=1985).

## Discussion

Physicians ordered the analgesics at 4-hour or 6-hour intervals, but the nurses' administration times varied widely. Among our patients recovering from cesarean delivery (i.e., matching Forkin et al. population) [1], the reality was a 289-minute interval of lateness (i.e., 95% of medication doses were administered between 108 minutes early and 181 minutes late). These were the times for the scheduled doses (i.e., not time waiting for "as needed" administration). A strength of our article was that, by being retrospective but using barcode scanning data, no bias was introduced through observation.

The results are important for the design of clinical trials of analgesic administration. The analgesic relevance is unknown because the observed benefit of the concurrent administration of acetaminophen and NSAID was likely a pharmacodynamic effect [1]. Forkin et al. (appropriately) had neither the word "concentration" nor "pharmacokinetic" in their article [1]. We likewise lacked the rationale for mathematically treating our Figure 1 as a window function to be convoluted with the estimated concentration over time curve for acetaminophen or ibuprofen. Nevertheless, just as ±30 minutes for time-critical medications [5,6] is a 1-hour interval, we observed a 4.8-hour interval for medications scheduled to be administered at 4-hour or 6‑hour intervals.

One limitation is that we found no factor to be an important predictor of lateness of analgesic administration among obstetric patients (Table 1). For example, time of the day and weekday, which may serve as proxies for variability in census and staffing, lacked important association with lateness. The lag-1 correlation was statistically significant, as expected, but so tiny (Kendall's tau = 0.014) as irrelevant. Further, the counts of overall medications to be administered among the patients at a given time, which could reasonably be expected to affect administration timing, were also not associated with lateness. Therefore, future work is needed to examine multiple specialties, hospitals, and health systems to learn how organizational culture influences the lateness of medication administration. Our control of antibiotic administrations suggests that the principal factor will not be the medication category.

Another limitation is that our results use data from one hospital [6]. However, the observation is novel based on PubMed, Scopus, and Google Scholar searches showing no earlier reports on the tardiness (i.e., lateness greater than zero) or earliness (lateness negatively valued) of medication administration postoperatively (https://FDshort.com/ItukDexterHadlandsmyth).

## Conclusions

Medication administration records revealed that 95% of analgesic doses were given between 108 minutes early and 181 minutes (i.e., 4.8-hour window), not ±30 minutes of the due time. We recommend that other hospitals check the lateness of administration of analgesics when that would change their ability to perform or apply the results of analgesic clinical trials (e.g., simultaneous versus alternating administration).
